# Smartphone delivery of a hope intervention: Another way to flourish

**DOI:** 10.1371/journal.pone.0197930

**Published:** 2018-06-01

**Authors:** Douglas A. Daugherty, Jason D. Runyan, Timothy A. Steenbergh, Betty Jane Fratzke, Brian N. Fry, Emma Westra

**Affiliations:** 1 Psychology Department, Behavioral Sciences Division, Indiana Wesleyan University, Marion, Indiana, United States of America; 2 LifeData, LLC, Marion, Indiana, United States of America; 3 Lumen Research Institute, Excelsia College, Sydney, Australia; 4 Sociology Department, Marion, Indiana, United States of America; University of Akron, UNITED STATES

## Abstract

Positive interventions have shown promise for fostering hedonic (happiness) and eudaimonic (flourishing) well-being. However, few studies have focused on positive interventions that target hope as a means of increasing well-being, and none have examined the use of smartphone app-based systems for delivering interventions in the moments and contexts of daily life—an approach called ecological momentary intervention (EMI). We conducted a quasi-experimental pilot study using a pretest and posttest design to examine the feasibility and potential impact of a mobile app-based hope EMI. Participants appeared to engage with the intervention and found the experience to be user-friendly, helpful, and enjoyable. Relative to the control group, those receiving the intervention demonstrated significantly greater increases in hope; however, there were no between-group differences in hedonic and eudaimonic well-being. The authors recommend future research to examine the potential of EMI mobile apps to cultivate hope and promote flourishing.

## Introduction

What does it mean to have hope? According to Snyder [1], [2], hope can be described as a positive motivational state resulting from having the sense that a good outcome will be reached, and that there are effective routes toward that outcome. For Snyder, hoping involves devoting will-power (e.g., agency, determination, commitment) and way-power (i.e., means to achieving an aim) toward reaching goals under conditions of intermediate to low probability [1], [2]. Hope is a position one has in relation to goals; it is “the sum of mental will-power and way-power one has for one’s goals” [1]. Hope comes into play when the achievement of a goal isn’t perceived as being either a slam-dunk or impossible [2].

### Hope, happiness & flourishing

Hope is encouraged by many world religions and philosophies [3], [4], [5], and has long been thought to promote eudaimonic well-being (EWB; e.g., Aristotle, Aquinas), [6], [1], or flourishing, analyzed in terms of having life purpose, meaning, and a sense of contributing to society [7]. It has also been thought to promote hedonic well-being (HWB), or happiness, analyzed in terms of experiencing pleasure and enjoyment [7].

A number of studies support these connections between hope and both forms of well-being. For example, it has been observed that hope predicts general well-being independent of both optimism and self-efficacy [8]. Additionally, hope has been shown to mediate the relationship between life purpose and life satisfaction in adolescents, emerging adults and adults [9]. Further, hope has been associated with positive affect among high-school students [10], and HWB among undergraduates [11]. Increased state well-being, and decreased state anxiety, have also been associated with hope in older adults [12].

There has, however, been some indication that hope has a more nuanced relationship with HWB. Frederick and Lowenstein [13] have theorized that HWB sometimes requires a particular form of adaptation, which involves accepting that, in certain situations, hope is lost. Experiencing HWB may involve admitting and coming to terms with loss; for instance, it might involve accepting the death of a missing family member [13]. Consistent with this theory, studies have shown that those willing to be tested for disease and notified of adverse test results—i.e., those willing to come to terms with loss in the form of an adverse diagnosis—often have greater HWB than those unwilling to be notified [14], [15].

### Positive interventions & hope

Examining ways of fostering EWB and HWB has been a central focus of positive psychology [16]. To this end, over the past 10 years a number of studies have examined the effects of positive interventions aimed at fostering well-being [17]. Many of these interventions have been delivered through the internet or computer, and have had positive effects on well-being These positive interventions have incorporated various strategies, with promising results, including practicing gratitude [18], [19], identifying and employing character strengths [18], [19], [20], reflecting on positive life events [21], goal-setting, and hope [22]. For example, gratitude interventions have been associated with positive relationships, enhanced physical health and greater eudemonic well-being [23]. The positive psychology literature in general, and the work of Charles Snyder in particular, suggests that hope interventions may be a promising means of enhancing well-being in the general population [18], [1], [2]. Therefore, targeting hope via smartphone delivery may be a means of promoting well-being and human flourishing.

With particular regard to hope, group psychoeducational interventions have been found to decrease anxiety, and increase life meaning and self-esteem [24]. Additionally, moving from hopelessness to hopefulness has been observed to facilitate substance abuse recovery [25]. There is also some evidence that hope interventions can have a positive effect [26], even if these benefits dissipate over time. Taken together, these initial findings suggest hope-centered interventions may promote well-being among those who desire change. The degree to which particular change is valued by the individual is worth highlighting as an important factor influencing well-being. Desire and readiness appear essential to realizing the benefits (i.e. enhanced well-being) associated with positive interventions, as suggested by the work of Lyubomirsky and colleagues [19] as well as Prochaska and DiClemente [27].

### Ecological momentary intervention

The past several years have witnessed a growing interest in the use of ecological momentary intervention (EMI) for the purpose of promoting well-being and its contributors [28], [29], [30]. EMI involves repeatedly intervening within the moments and context of daily life. And this approach has several advantages over traditional computer or internet interventions.

First, EMI promotes self-monitoring and self-awareness throughout the day, which has been shown to foster positive change when an individual is motivated and able to change [31], [32], [33], [34]. Second, EMI can be used to prompt individuals to redirect their thoughts and attention, or engage in practices and activities, repeatedly throughout the various contexts of daily life; and doing things repeatedly in multiple contexts can foster the development of more stable habits or dispositions [35], [36], [37]. Context can act as an “occasion-setter” by making it more likely a person will respond as they have repeatedly done in that context, and similar contexts, in the past [38], [39], [40], [41].

Over the past five years, advances in smartphone technology, and the near ubiquitous integration of this technology into everyday life, has made EMI more practical for widespread use [17], [42]. In particular, flexible mobile app-based platforms are making the design and widespread dissemination of EMIs quick, user-friendly and cost-effective (e.g., LifeData, Metricwire, Ilumivu, Movisens). This is making it increasingly feasible to conduct EMIs with devices that an ever-growing number of people already use throughout the day [43]. Smartphones are, thus, making it increasingly easier to deliver and scale EMIs.

### Study aims

In this quasi-experimental, pilot study, our primary aim was to conduct a feasibility study of a smartphone based EMI designed to foster hope and thereby promote well-being. Feasibility studies are indicated when few studies or data are available on a specific intervention technique and help researchers determine whether the intervention is appropriate for further testing [44]. Our secondary aim was to examine whether a hope EMI—designed and disseminated using a flexible mobile app-based system—increased hope, HWB and/or EWB. Studying the feasibility of positive mobile app-based EMIs, such as the one described here, is timely given the extent to which smartphones are making these interventions practical and scalable. EMIs are also more mobile than traditional computer or internet interventions, enabling interaction with people in the moments and contexts of everyday life. To our knowledge, this is the first study to examine a hope-focused EMI.

## Methods

This study was reviewed and approved by the Indiana Wesleyan University Institutional Review Board prior to the initiation of the study.

### Participants

We used a convenience sample of residential undergraduate volunteers drawn from behavioral science classes at Indiana Wesleyan University, a religiously-affiliated liberal arts university in Marion, IN. Seven classes were randomly assigned to the intervention or control conditions. Students in four classes (n = 66) were assigned to the intervention condition and three classes (n = 46) served as controls. Of the 112 participants, 79 (70.5%) were female, and 99 (88.4%) identified as White Non-Hispanic. There was a fairly even distribution of freshmen, sophomores, juniors and seniors. Although participants were not asked to provide their age, the range for all participants was 18–25 years, as this was a sample of traditional-age college students. Participants in both groups were offered extra credit for participating in the study. Only 16 of 46 control participants completed the post-test compared to 41 of 66 EMI participants.

### Measures

#### Snyder hope scale

The Snyder Hope scale was used to assess pretest and posttest hope. This scale consists of 12 items and respondents rate each item on a 4-point scale (1–4). Four items assess will-power, four items assess way-power, and four items serve as distractor items [45]. For the purposes of this study, the four distractor items were omitted because they do not assess the Hope construct and would have increased response demand. Due to a programming error, one of the will-power items (“My past experiences have prepared me well for the future”) was not received by study participants. As a result, will-power was assessed with only three items. Will-power, way-power and total Hope scores were calculated by summing items for the respective scales. Previous studies have demonstrated that the Snyder Hope scale has sufficient internal consistency, with Cronbach's alpha ranging from .74 to .78 in various samples [8]. Temporal stability has been evidenced by test-retest correlations of .73 to .82 [8], [45]. In this study, the measure demonstrated adequate reliability for the seven-item general hope index (α = .86), the four-item way-power subscale (α = .82), and the three items from the will-power scale (α = .70). Evidence for the construct validity of the Hope Scale has been suggested by observing that higher scorers, relative to lower scorers, have more goals and goal-achievement, better coping skills, less distress and better recovery from physical injury [46].

#### Steen Happiness Index (SHI)

The SHI was used to assess pretest and posttest HWB. It was developed as a measure of happiness, or HWB, and consists of 20 items [18]. For each item, participants select one of five statements that best describes them at present. Each response is assigned a value ranging from 1 to 5, with 5 always indicating the happiest response. Total scores are calculated by summing assigned values to all items. The total score is the sum of these items, with a possible range of 20 to 100 for the scale [18]. The scale has demonstrated strong correlations with other happiness measures, but provides a more normal distribution of scores, relative to other happiness measures [18]. In the present study, we observed a high degree of internal consistency among SHI items (α = .93).

#### Flourishing scale

The Flourishing Scale consists of eight items intended to measure EWB. Each item is scored on a 7-point scale (1–7) and the sum of all eight items provides an overall flourishing score. The measure has strong internal reliability as evidenced in this study (α = .88) and previous research [47].

#### App engagement

App engagement was measured by whether the participant responded to notifications within the app. Responding was operationalized as the number of times that participant tapped on a notification or initiated the always available content, viewing at least one screen in either case. All such interactions were tracked by the app and timestamps were gathered to confirm participant engagement.

#### Questions about the app-intervention experience

Intervention participants were asked, in-app at posttest, about their experience with the app intervention. The questions, listed below, based on the researchers previous experience with other ecological momentary assessment (EMA) and EMI studies [28], [33], utilized five 7-point Likert-type scale items (1–7) with anchors of “not at all” and “extremely”, and included two open-ended items at the end.

1. To what extent have you had technical difficulty with the app?
2. How easy was the app to use?
3. How likely would you be to use this app again?
4. Was using the app enjoyable?
5. To what degree did you find this app helpful?
6. In what ways did the app help you?
7. What suggestions would you make for improvements?

#### Ecological momentary intervention

The hope EMI used for this study was designed and disseminated using the LifeData System, a flexible ecological momentary assessment and intervention mobile app-based platform (www.lifedatacorp.com). We used this system to deliver random in-the-moment hope notifications. These notifications—analogous to text message notifications—either caused participants’ phones to vibrate, ding, or both, depending upon their phone settings. A banner would also pop up on their screen indicating they had a hope intervention waiting on them. Swiping on the banner took participants to the first hope prompt.

With each in-the-moment hope notification, participants received three types of prompts, in this order: (1) a hope picture-statement (e.g., a Nelson Mandela quote on a tinted background, a mountain scene with a statement expressing hope, etc.); (2) a hope statement (e.g., hopeful inspirational statement, hopeful spiritual statement); and (3) one of three questions drawn from the Snyder Hope scale (e.g. “At the moment, I can think of many ways to get out of a jam”) with response options ranging from definitely false to definitely true. The hope stories were solicited from university students who were asked to “share a story of hope.” The messages followed from a Google search of “hope messages” as well as “hope quotes” and “hope scripture” with consensus among the lab team (researchers) determining final selection of the content. Here is one hope story example:

“In 2011, I had to undergo a major surgery where they reconstructed my bones. After that surgery, I essentially had to learn how to walk all over again. That surgery caused me an immense amount of emotional distress, but going through that process taught me a lot about persistence and perseverance. Following the surgery, I dealt and still deal with much insecurity about my body. However, recently I have found hope in my ability to love my body the way that God created it to be.”

These peer stories of hope were always available to participants. In other words, these 10 stories could be accessed at any time by opening the app and selecting a story.

### Procedure

After signing a consent form, participants downloaded the free LifeData smartphone app, RealLife Exp, from the Apple App Store or Google Play. Each class then downloaded either the intervention or control study protocol through the mobile app. To start the study, participants in both groups completed demographic questions and baseline assessments of hope, HWB and EWB through the mobile app.

After completing the initial assessments, participants in the intervention group received a 28-day mobile app intervention that included 1 to 3 random, in-the-moment hope notifications delivered Monday through Saturday and the always available peer stories of hope.

Participants in the control condition completed the same initial assessment with the app as those in the intervention condition. They did not, however, receive any random notifications or have access to the peer stories of hope in the app control condition. On days 29 through 31, participants in both groups received in-app notifications prompting them to complete the posttest measures.

### Statistical analyses

All statistical analyses were conducted with SPSS v.23 and Type I error rates were set at p = .05 due to the small sample size and exploratory nature of this pilot study. Scale scores were calculated as described above. Skipped items resulted in some individuals not having a score on the scale to which the skipped item belonged. To examine whether intervention and control groups differed on continuous variables, two-tailed independent samples t-tests were conducted. Chi-square was used to compare distributions of categorical variables. Pearson product-moment correlation coefficients tested associations between dependent variables. One-tailed correlations were calculated due to the positive associations that are typically been found between these variables. Those that were significant were used as covariates for repeated measures ANOVA. Repeated measures ANOVAs assessed differences in pretest and posttest assessments across the intervention and control groups. Partial η^2^ was calculated to estimate effect sizes [48].

## Results

On average, participants in the intervention group responded to 83.5 (*SD* = 40.1) of the total 135 notifications delivered, constituting a response rate of 61.9%. They also initiated engagement with peer stories of hope an average of 19.1 (*SD* = 16.1) times over the course of the 4-week intervention. The study-wise posttest response rate was 50.9%; however, these rates varied considerably across conditions: 41 of 66 (62.1%) intervention participants vs. 16 of 46 (34.8%) control participants.

### Pretest differences and associations

There were no significant differences between the intervention and control group in terms of gender, χ^2^(2) = .77, *p*>.05, or ethnicity, χ^2^(4) = 1.18, *p*>.05. Pretest HWB, EWB, Hope, Way-power and Will-power scores for intervention and control groups are presented in Table 1 and further demonstrate similarities between groups at pretest. Table 2 presents the zero-order correlations between the pretest and posttest wellbeing and hope measures. Additional independent samples t-tests examining posttest HWB, EWB, and Hope scores revealed no significant differences between groups on gender or minority status (*p*’s>.05); therefore, these were not used as covariates in later repeated measures ANOVAs.

**Table 1:** Unadjusted pretest mean (and SD) Scores for HWB, EWB and hope scales by condition.

Measure	Intervention	Control	*Df*	*t*	*p*
HWB	57.12 (11.12)	56.31 (12.99)	97	.33	.74
EWB	42.46 (7.13)	44.30 (5.08)	107	-1.47	.14
Hope	20.50 (3.12)	20.47 (3.46)	103	.05	.96
Will-power	8.87 (1.37)	8.86 (1.55)	103	.04	.97
Way-power	12.22 (1.62)	11.74 (2.22)	110	-07	.95

Note: Possible scores could range from 20–100 for HWB, 8–56 for EWB, 7–28 for Hope, 3–12 for Will-power, and 4–16 for Way-power.

**Table 2:** Correlations among pretest and posttest wellbeing and hope measures.

		Pretest					Posttest				
		HWB	EWB	Hope	Way	Will	HWB	EWB	Hope	Way	Will
	HWB	1.0 (99)	.66*** (96)	.70*** (99)	.60*** (99)	.72*** (97)	.74*** (50)	.58*** (50)	.34** (51)	.22 (51)	.45** (51)
	EWB		1.0 (109)	.45*** (102)	.32*** (109)	.51*** (102)	.44*** (53)	.63*** (55)	.24* (54)	.12 (56)	.36** (54)
Pretest	Hope			1.0 (105)	.89*** (105)	.95*** (105)	.54*** (50)	.50*** (52)	.62*** (52)	.55*** (53)	.61*** (52)
	Way				1.0 (112)	.69*** (105)	.52*** (54)	.47*** (56)	.54*** (55)	.50*** (57)	.50*** (55)
	Will					1.0 (105)	.36** (50)	.34** (52)	.51*** (52)	.43** (53)	.53** (52)
	HWB						1.0 (54)	.71*** (53)	.59*** (53)	.49*** (54)	.64*** (53)
	EWB							1.0 (56)	.64*** (54)	.54*** (56)	.63*** (54)
Posttest	Hope								1.0 (55)	.95*** (55)	.88*** (55)
	Way									1.0 (57)	.69*** (55)
	Will										1.0 (55)

Notes: Sample sizes are presented in parentheses.

*p < .05

**p < .01

***p < .001

### Effects of intervention on hope

**Fig 1:**
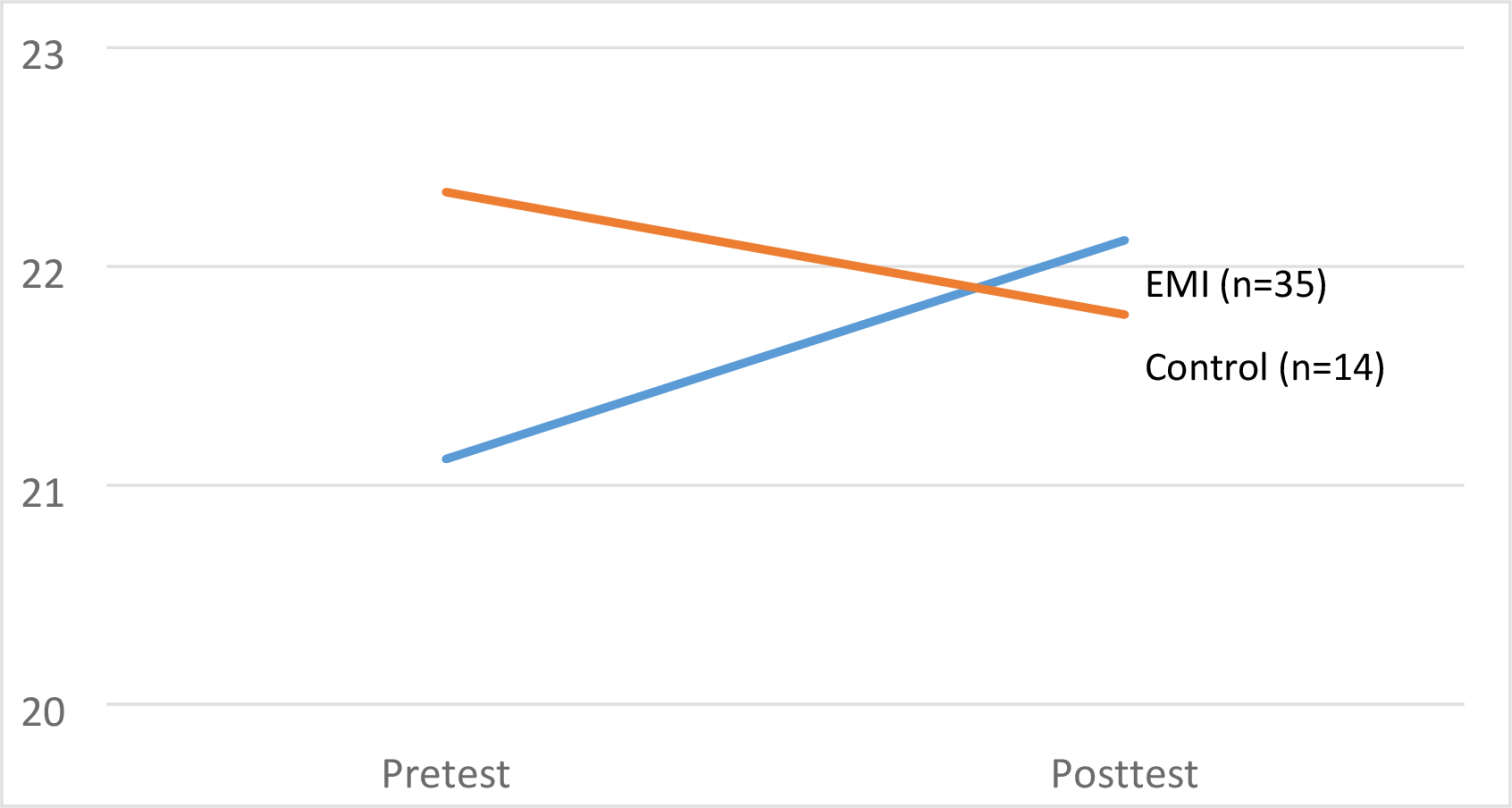
Estimated marginal means for hope scores by condition. Notes: Estimated marginal means are adjusted for pretest EWB and HWB.

To test whether there were significant changes in hope across the intervention and control conditions, a repeated measures ANOVA was conducted. Condition was the between groups variable and total Hope scores at pretest and posttest were the repeated measures. Because pretest EWB and HWB were significantly correlated with posttest Hope scores, these were used as covariates. Results revealed a significant interaction effect, *F*(1,45) = 4.24, *p* = .05, which is illustrated in Fig 1. The estimated marginal mean increase in pretest Hope scores among those in the intervention group (+1.00) was significantly greater than that observed in the control group (-.56), and this constituted a moderate effect size, partial η^2^ = .089.

Similar repeated measures ANOVAs for the Will-power and Way-power subscales were run. Because posttest Will-power scores were correlated with pretest HWB and EWB scores, these were used as covariates. Results revealed no condition (EMI vs control) by time (pretest to posttest) interaction effect, *F*(1,45) = 2.60, *p* = .11; partial η^2^ = .055. Because Way-power scores were not significantly correlated with pretest scores, covariates were not used in the third repeated measures ANOVA. A condition by time interaction effect was also not found for Way-power scores, *F*(1,45) = 2.14, *p* = .15; partial η^2^ = .037. Small effect size, combined with small sample sizes to limit the statistical power for both the Will-power and Way-power analyses (*Power coefficients were* .35 and .30, respectively).

### Changes in HWB and EWB

Two additional repeated measures ANOVAs tested whether there was an increase in HWB and EWB for the EMI group, relative to the control group. For both analyses, condition served as the between groups factor and pretest and posttest HWB and EWB scores were the respective repeated measures. EWB and Hope scores were used as covariates in the analysis of HWB scores, and results revealed no condition by time interaction effect, *F*(1,44) = .22, *p* = .64, η^2^ = .005. The statistical power for the analysis was very low at .08. Analysis of EWB scores, with HWB and Hope scores as covariates, also revealed no interaction effect, *F*(1,44) = 1.62, *p* = .21, η^2^ = .036. The statistical power for that analysis was .24.

### App engagement and acceptability

In the intervention(EMI) group, level of app engagement (i.e., the number of times participants responded to notifications or accessed always available peer stories) was not significantly related to posttest HWB, overall Hope, Will-power, Way-power, or EWB (*p’*s>.10). However, app engagement was significantly related to pretest measures of total Hope, *r*(60) = .27, *p* = .03, and Way-power, *r*(64) = .27, *p* = .03. Total app engagement for females (*M* = 114.1, *SD* = 37.2) was significantly higher than males (*M* = 72.4, *SD* = 47.4), *t*(62) = 3.62, *p* = .001. There was also a trend for greater engagement among minorities (*M* = 135, *SD* = 35.8), relative to non-minorities (*M* = 107.2, *SD* = 39.3), *t*(55) = 1.77, *p* = .08. The small number of minority participants (*n* = 7) resulted in low statistical power for this comparison.

At the end of the study, participants in the intervention condition provided feedback regarding their experience using the mobile app intervention. Table 3 presents mean responses on a 7-point scale (i.e., 1-not at all to 7-extremely) and suggests that, in general, participants found the app intervention helpful, enjoyable, and easy to use. Participants also indicated having minimal technical difficulties. A representative sample of open-ended comments that were solicited from participants is provided in Table 4. Of the 27 comments provided, 93% (*n* = 25) were positive and 7% (*n* = 2) were negative. The two negative comments were: “It didn’t help me. I feel much worse than I did at the beginning.” and “It didn’t help me much.”

**Table 3:** Mean and SD for feedback about app experience.

Question	*n*	*M*	*SD*
Easy to Use	41	5.88	1.00
Use Again	42	4.48	1.77
Enjoyable to Use	42	4.29	1.25
Helpful App	40	4.08	1.46
Technical Difficulties	38	2.58	1.83

**Table 4:** Participants’ comments about the app.

Helped me with daily encouragement when needed
I appreciated how easy it was to use.
I loved reading the encouraging messages.
I enjoyed seeing the messages of hope every so often
It reminded me to stay positive throughout the day
The app was actually pretty encouraging in everyday tasks
It made me think about how hope is integrated into my life
It helped me reflect upon myself

## Discussion

Our findings suggest that mobile app-based EMIs may provide a feasible means of fostering hope in the moments of daily life. The study intervention targeted hope via in-the-moment notifications, and peer stories of hope that could be accessed at will. The intervention group demonstrated a significant increase in overall hope from pretest to posttest assessment, relative to the control group; however, associated group differences in EWB and HWB were not observed. Intervention participants viewed peer stories of hope repeatedly throughout the 4-week intervention without prompting or extrinsic incentive. Further, the app experience was met with favorable feedback ratings and positive open-ended comments. Unfortunately, the absence of an active control condition, in which participants were regularly interacting with the app, greatly limited engagement, including posttest engagement, among control participants.

### Intervention effects

Those in the hope intervention experienced a significant increase in hope from pretest to posttest, and the effect size for the intervention was moderate. While this provides some evidence for the impact of a hope-focused EMI, without an active control condition we cannot rule out the possibility that this effect may have resulted from the interactive experience itself and had less to do with hope per se. With that said, however, we cannot identify a theoretical explanation for why interacting with an app throughout the day would impact hope unless the content of that interaction itself was hope-inspiring.

We expected to find that increases in hope among those in the intervention group would be accompanied by an increase in HWB or EWB. Our hypothesis was based on previous work examining the positive relationship between these variables [7], [6], [1]. Further, the correlational findings from this study support the positive relationship between hope, HWB and EWB. It may be that the magnitude of the hope effect we observed was insufficient to bolster posttest HWB and EWB. Posttest hope scores were moderately correlated with posttest HWB (.59) and EWB (.64), accounting for 35% and 40% of the variance in these measures, respectively. Therefore, given the moderate increase in hope and the moderate relationship between hope and our measures of wellbeing, it may not be surprising that significant effects on HWB and EWB were not observed.

Other reasons for the lack of concordance between increasing hope scores and resulting scores on HWB and EWB should be considered. Given the nature of this feasibility study, it would seem that the most plausible explanation involves the low statistical power to detect differences in this study. However, it is important to recognize that the correlational nature of most studies on hope and well-being preclude us from ruling out the possibility of a third variable explanation [49]. Further, given the fact that at least one other study has failed to demonstrate a lasting effect on EWB following a brief intervention that successfully increased hope [26], additional study of the mediational role of hope on HWB and EWB seems warranted.

### EMI feasibility and engagement

Data gathered from intervention participants who answered questions about their experience with the mobile app at the end of the study suggested that the hope EMI was well-received. Participants reported that the app was easy to use and that they had few technical difficulties. Overall ratings suggested a tendency for participants to report they would use the app again, and that they found it helpful and enjoyable. However, these ratings were not overwhelmingly positive and support the need for ongoing work in developing a robust, engaging mobile application experience.

Feasibility of mobile interventions, like the one discussed here, can be considered from several viewpoints, including those of researchers, clinicians, patients and others. The ubiquity of smartphones makes in-the-moment interventions accessible and cost-effective in clinical and public health contexts, although challenges remain in translating empirically-supported face-to-face interventions for mobile delivery. The current study made use of web-based software to build and deliver content by means of a mobile app. This system, and others like it, continue to evolve—making it increasingly easy to create and modify EMI content, notifications and experiences. From a participant’s perspective, these interventions are easily embedded in everyday life, with potential for offering desired “coaching” and support in-time, on-time and over-time. Further advances in the science of EMI will be needed if these positive interventions are to be consistently engaging, motivating, and helpful.

Findings from this study should also be considered in light of the participant demographic. College students spend a great deal of time each day on their smartphones for a variety of activities, including school work [50]. With over 90% of college-age students now owning a smartphone [51], and some data suggesting that they may spend over 8 hours per day on their smartphone [52], this represents a particularly promising but challenging demographic. On one hand, college students are especially adept at using smartphones; however, their smartphones represent very competitive environments where users spend much of their time on social activities like texting, Facebook, Twitter and Instagram [52]. Increasingly, mobile developers need to recognize the importance of “competing” with other applications that users regularly engage. Adding interactive and social components may hold promise in this respect.

### Limitations and future directions

While the hope EMI appeared to engage participants and lead to increased levels of hope, several study limitations demonstrate the need for additional investigation in this area. Chief among these limitations was the study design. Because classes and not participants were randomized to conditions, we cannot rule out the possibility that other variables, outside those we measured were responsible for the changes we observed in hope. Another significant design issue involved the use of an inactive control group [21]. As alluded to earlier, those in the control group did not engage with the app over the course of the study, so we cannot disentangle possible effects related to interacting with the app from those specific to the hope intervention itself. Furthermore, we believe that the lack of engagement with control participants throughout the course of the study substantially contributed to their low rate of posttest completion.

The current study was a pilot, intended to examine the feasibility of mobile hope interventions. As Leon, Davis and Kraemer [53] discuss in their seminal work on the subject, pilot studies are intended to examine feasibility rather than distal outcomes (effectiveness). Replication of the current study with a larger, more diverse sample using an experimental design would be a reasonable next step.

Our study was conducted at a religiously-affiliated liberal arts college. While this enabled us to use religious statements to encourage hope in our intervention, future studies might examine whether similar or altered hope interventions—not including hopeful religious statements—have a similar effect in a non-religious context. This might be done by drawing from the general population, including and, perhaps, prioritizing disadvantaged and underserved groups. In fact, we think that smartphone interventions may be especially well-suited to extending the reach of positive psychology to include underserved groups [28]. National data suggest that cell phone ownership among minorities is high [43] and this, coupled with some evidence for higher rates of engagement among minorities in this study suggest that such interventions hold promise.

Future studies might examine the potential merits of positive smartphone interventions focused on clinical populations as well. Seligman [16] and Clough and Casey [54], among others [55], [56], [57], [42], [58], [59], [60], provide a useful overview of the possibilities and challenges for positive interventions with clinical populations. These studies might also explore the value of smartphone interventions that target a combination of strengths/virtues (e.g. hope and gratitude) delivered over time and in the context of daily life. This is consistent with virtue theory that suggests that it is the combination of virtues that lead to flourishing [61], [62], [63].

Future directions for research might include the use of mobile “just-in-time adaptive interventions” (JITAI) to foster hope. Nahum-Shani and colleagues [64] have discussed the merits and challenges associated with providing just the right intervention, at the right time, to foster attitudinal and behavioral change. The authors offer a conceptual framework and key principles for JITAI interventions. Future research might employ mobile hope, just-in-time interventions that adapt to the dynamic responses and/or particular situations of end-users.
